# Modelling excitation energy transfer and trapping in the filamentous cyanobacterium *Anabaena variabilis* PCC 7120

**DOI:** 10.1007/s11120-020-00723-0

**Published:** 2020-02-19

**Authors:** Avratanu Biswas, Xinpeng Huang, Petar H. Lambrev, Ivo H. M. van Stokkum

**Affiliations:** 1 Department of Physics and Astronomy and LaserLaB, Faculty of Science, Vrije Universiteit Amsterdam, De Boelelaan 1081, 1081 HV Amsterdam, The Netherlands; 2 Biological Research Centre, Szeged, Temesvári krt. 62, Szeged, 6726 Hungary

**Keywords:** Global analysis, Target analysis, Light harvesting, Phycobilisome, Photosystem I, Photosystem II

## Abstract

The phycobilisome (PBS) serves as the major light-harvesting system, funnelling excitation energy to both photosystems (PS) in cyanobacteria and red algae. The picosecond kinetics involving the excitation energy transfer has been studied within the isolated systems and intact filaments of the cyanobacterium *Anabaena variabilis* PCC 7120. A target model is proposed which resolves the dynamics of the different chromophore groups. The energy transfer rate of 8.5 ± 1.0/ns from the rod to the core is the rate-limiting step, both in vivo and in vitro. The PBS-PSI-PSII supercomplex reveals efficient excitation energy migration from the low-energy allophycocyanin, which is the terminal emitter, in the PBS core to the chlorophyll *a* in the photosystems. The terminal emitter of the phycobilisome transfers energy to both PSI and PSII with a rate of 50 ± 10/ns, equally distributing the solar energy to both photosystems. Finally, the excitation energy is trapped by charge separation in the photosystems with trapping rates estimated to be 56 ± 6/ns in PSI and 14 ± 2/ns in PSII.

**Electronic supplementary material:**

The online version of this article (10.1007/s11120-020-00723-0) contains supplementary material, which is available to authorized users.

## Introduction

**Fig. 1:**
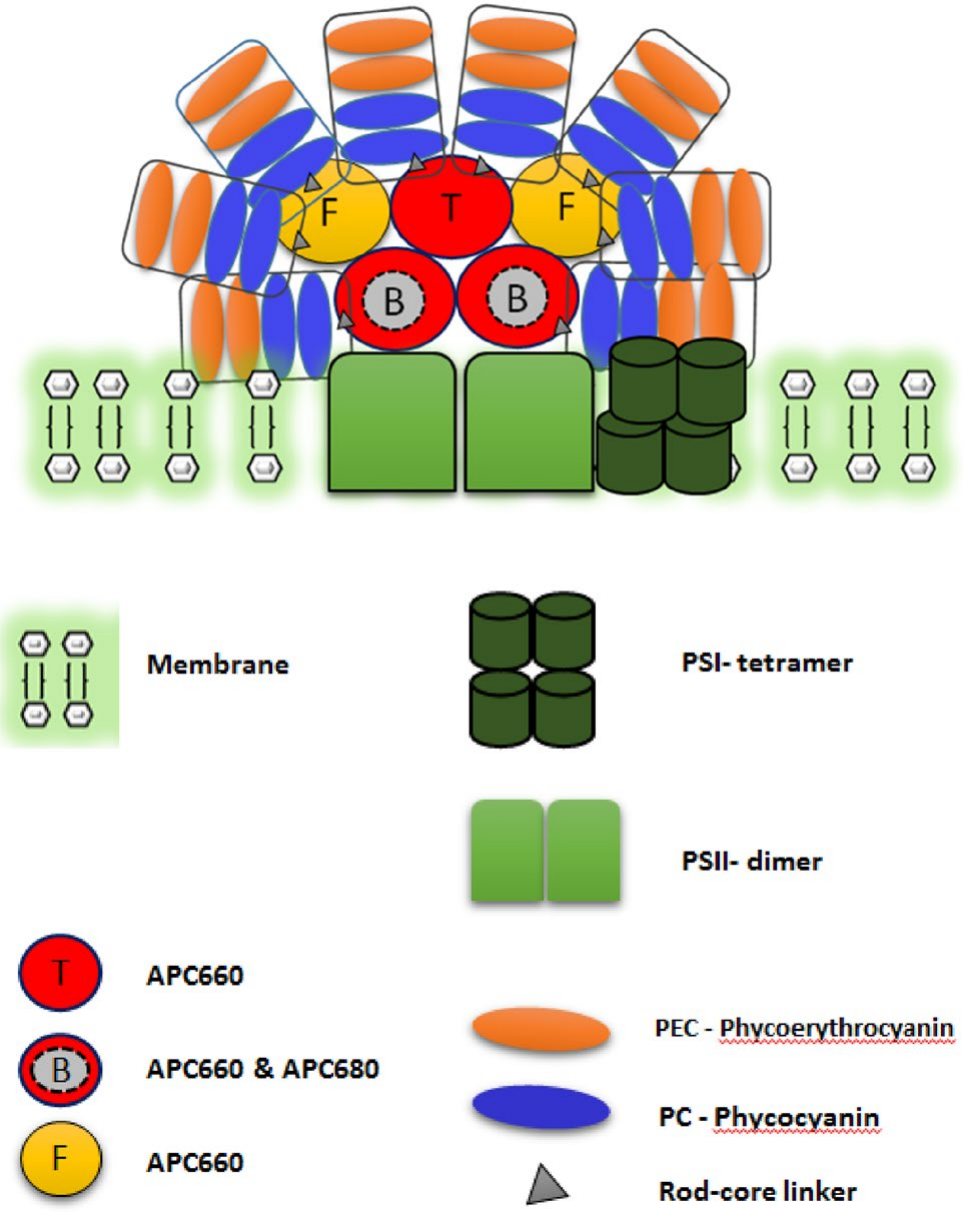
Schematic representation of the penta-cylindrical PBS structure with two basal cylinders (B), one top cylinder (T) and two flanking cylinders (F) of *Anabaena *proposed by Ducret et al. (1998). The direct physical interaction of the PBS to the PSII dimer has been shown before in Liu et al. (2013), Acuna et al. (2018b). The schematic representation further shows the supercomplex of PBS-PSII-PSI in which PSI in *Anabaena* is present in a tetrameric form as reported in Watanabe et al. (2011,2014)

Photosynthesis is initiated by a network of light-absorbing chromophores capturing the sunlight and funnelling the excitation energy from one light-harvesting complex to another and finally getting trapped at a photochemical reaction centre (RC), where charge separation takes place. The two distinct RCs are the P700 and P680 of photosystem I (PSI) and photosystem II (PSII), respectively, in oxygenic photosynthetic organisms such as cyanobacteria. Unlike in higher plants where the light-harvesting complex is a macromolecular complex primarily consisting of Chl*a*, Chl*b* and chlorophyll*a/b* binding proteins (Cab), the light-harvesting complex in cyanobacteria, red algae and glaucophytes is composed of a group of water-soluble pigmented proteins, called phycobiliproteins, that together form a supramolecular structure called the phycobilisome (PBS) (Grossman et al. 1993; Bar-Eyal et al. 2018). The PBS is attached to the cytoplasmic side of the thylakoid membrane, and has a hemi-discoidal shape in cyanobacteria with a central core from which 4–8 rods radiate out. The major building block of PBS is a heterodimer of α and β sub-units. These sub-units assemble in trimers (αβ)_3_ or hexamers (αβ)_6_ to form disc-like structures, which further stack together to form cylinders (Arteni et al. 2009; MacColl 2004). The number of cylinders in the core of the PBS varies within the cyanobacterial species. Electron microscopy (EM) studies of *Synechocystis *sp. (Elmorjani et al. 1986; Arteni et al. 2009) have revealed two cylinders lying on the thylakoid membrane (basal cylinders) and a top cylinder with a C_2_ rotational symmetry. Each cylinder is a stack of four trimers (discs) with each disc containing six APC pigments. These APC pigments can be APC_660_ (*λ*_max_ of emission 660 nm) and APC_680_ (terminal emitter) pigments. The topmost cylinder consists only of APC_660_ pigments. The discs within each cylinder have different polypeptide composition. In contrast, the PBS of *Anabaena variabilis* PCC 7120 (hereafter called *Anabaena*) belongs to the same hemi-discoidal family but contains a penta-cylindrical core (Ducret et al. 1998; Glauser et al. 1992). The APC core resembles the tri-cylindrical core of *Synechocystis* with two supplementary half-cylinders, flanking the other core cylinders, cf. Fig. 1. The ultra-structural organization of the PBS in *Anabaena* is yet to be studied in detail. The peripheral rods radiating from the core constitute phycocyanin (*λ*_max_ = 620 nm) as the major pigment for the rods adjacent to the core, and phycoerythrocyanin (PEC, *λ*_max_ = 575 nm), are found at the core-distal ends of the peripheral rods depending on the growth conditions (Glauser et al. 1992). The assembly of discs into cylinders, rods and the connection between the rods and the core is facilitated by several types of colourless linker proteins. A cascade of excitation energy transfer (EET) takes place from the rod to the core. In principle the fastest energy transfer component dominates within the rods and the energy transfer process from the C-PC trimer to the APC core is considered as the rate-limiting step (Zhang et al. 1997). Recently, an elaborate functional compartmental model has been developed to describe the microscopic rates within the rods, from rod to core and within the core (van Stokkum et al. 2018). Each of these compartments contains many pigments. Ultimately, the challenge would be to connect these effective rates that describe EET between compartments to specific routes of EET in a PBS structure, like the recent cryo-EM PBS structure of the red algae, *Griffithsia pacifica* (Zhang et al. 2017).

PBS is connected to the membrane via a multidomain core-membrane linker, *L*_CM_ (Tang et al. 2015). Ultra-fast time-resolved spectroscopy measurements have been used to probe the rates of EET from PBS to photosystems (PSs) in cyanobacteria such as *Synechocystis* (Tian et al. 2011) and *Synechococcus* (Acuna et al. 2018a). The efficiency of light energy transferred from PBS to the chlorophylls (Chls) in PSI and PSII approaches 100% (Glazer 1984). Several works have established the energy migration evidences from PBS to PSI, for instances, there is efficient energy transfer from PBS to PSI in a *Synechocystis* mutant lacking PSII (Mullineaux 1994) and in *Anabaena* heterocysts, where PSII is also absent (Peterson et al. 1981). The close structural proximity of the PBS to PSII is convincing enough to establish a direct route of EET from PBS to PSII, whereas the exact route by which the energy is transferred from PBS to PSI and the microscopic rate involved has been yet a matter of debate. It is believed that the PBS feeds excitation energy to PSI via a direct interaction between them (Mullineaux 1992, 1994; Kondo et al. 2007). In fact, direct interaction of PBS rods with a PSI tetramer has been revealed recently by an electron microscopy study of the supercomplex isolated from *Anabaena *sp. PCC 7120 (Watanabe et al. 2014). It has been reported that in the case of *Anabaena*, the PSI-to-PSII ratio remains 6:1 in autotrophically grown cells (Mannan and Pakrasi 1993), which gradually increases in nitrogen-starved cells (Onishi et al. 2015).

In the present work, we performed time-resolved fluorescence measurements on whole *Anabaena* filaments as well as isolated PSI and PBS. We investigated the energy migration dynamics within these in vivo and in vitro systems. A bottom-up approach has been adopted where we acquire preliminary biophysical properties of the individual systems using target analysis of the individual systems and finally, with the help of simultaneous target analysis, we develop a functional compartmental model of the excitation energy transfer and trapping in *Anabaena* cells.

## Materials and methods

### Culture conditions

Cyanobacterial filaments were cultivated in liquid BG11 medium at room temperature (25 °C) supplemented with 5 mM HEPES–NaOH (pH 7.5) under continuous white light illumination of 50 umol photons PAR m^−2^ s^−1^. The flasks were shaken at a speed of 170 rpm.

### Sample preparations

#### Phycobilisomes

Phycobilisomes were prepared from *Anabaena* filaments according to Garnier et al. (1994) with some modifications. The optical density (OD) of the cultures taken for the isolation was between 0.7 and 0.9 OD_680_. The filaments were pelleted at 7000×*g* and washed with extraction buffer (with 1 mM protease inhibitor). The filament pellet was broken using 106 μm glass beads using a bead-beater homogenizer and was centrifuged at 3000×*g* for 5 min at 14 °C for pelleting down the filament debris. The supernatant was treated with triton X-100 (3%) for half an hour in the dark. The upper greenish layer was discarded and the sample was loaded onto a sucrose density step gradient of 1 M, 0.75 M, 0.5 M, 0.25 M sucrose in 0.75 M isolation buffer. The gradient was centrifuged for 16 h at 26,000 rpm, 14 °C. PBS containing fraction was collected and characterized by steady-state spectroscopy for further use.

#### Thylakoid extraction and PSI isolation

Thylakoid extraction was performed as described previously (Watanabe et al. 2011) with a few modifications. The filaments re-suspended in buffer A (20 mM MES, 10 mM MgCl_2_, 10 mM CaCl_2_, pH 6.5) were disrupted using glass beads. A cycle of 15 s agitation and 2 min cooling was repeated 15 times. Broken cells as pellets were removed and the supernatant was centrifuged at 15,000×*g* for 15 min at 4 °C to obtain thylakoid membranes as pellets. The pellet was re-suspended with buffer A supplemented with 25% glycerol for later use. For sucrose density gradient centrifugation, thylakoids were solubilized with 1% β-DM and incubated for 30 min in the dark at 4 °C followed by 15,000×*g* centrifugation at 4 °C. The supernatant was loaded on a 10–30% linear sucrose density gradient and centrifuged at 200,000×*g* for 16 h at 4 °C. PSI-enriched fraction was collected using syringe.

### Steady-state spectroscopy

Room temperature absorption spectra were recorded using a Varian Cary 4000 UV–Vis spectrophotometer. The absorption spectra were scanned in the visible region from 400 to 750 nm with 1 nm bandwidth using standard glass cuvettes with an optical path length of 1 cm. The fluorescence emission spectra were recorded using a Fluorolog 3.22 spectrofluorimeter (Jobin Yvon-Spex). For 77 K measurements, the same setup was used with an additional home-built liquid N_2_-cooled low-temperature device. The PSI-enriched sample was measured with an OD of ≈ 0.05 (diluted with buffer A) at the maximum of the *Q*_y_ absorption band with 400 nm excitation, detected in the 600–800 nm wavelength range. The isolated PBS was measured with 577 nm excitation with an OD_620_ ≈ 0.05 (diluted with 0.75 M phosphate buffer), detected within the spectral range between 600 and 720 nm. The spectral bandwidth was 5 nm and 2 nm for excitation and emission, respectively, with 1 s integration time.

### Time-resolved fluorescence measurements

The picosecond time-resolved measurements were performed using a synchroscan streak-camera setup as described previously (Wlodarczyk et al. 2016; Le Quiniou et al. 2015; van Stokkum et al. 2008). Briefly, the setup is comprised of a femtosecond laser source, Coherent Vitesse Duo, which pumps the regenerative Coherent RegA 900, and the output from the RegA further feeds to the optical parametric amplifier Coherent OPA 9400 (output wavelength from 470 to 770 nm). Vertically polarized emission was collected at a right angle to the incident beam by a spectrograph (Chromex 250IS, 50 grooves/mm ruling, blaze wavelength 600 nm, spectral width of each image is 260 nm). The central wavelength was set at 680 nm for all the experiments. Scattered excitation was removed with an optical long-pass filter. Further from the spectrograph, the light was focused on the input slit (40 μm) and further to the photo-cathode of the streak camera Hamamatsu C5680.

Two excitation wavelengths were used, 400 (for predominant Chls excitation) and 577 nm (for predominant PBS excitation) for the *Anabaena* filaments measurements, with a laser power set to 60 μW. The isolated samples (PBS and PSI) were measured with 400 nm excitation. The laser power was set to 90 μW and 100 μW for PBS and PSI, respectively. In all the cases, the laser beam was focused to a small spot of ≈ 50 μm diameter, with a laser repetition rate of 150 kHz. For the whole filament measurement, the sample was diluted to less than ≈ 1.5 at OD_680_ using the growing media BG-11, to avoid significant self-absorption and was magnetically stirred in a cuvette (1 × 1 × 4 cm^3^) with speed 750 rpm in order to avoid any photodamage of the sample. The intact cells sample was dark adapted for 20 min prior to the measurement, thus the intact cells are in state 2 (Dong and Zhao 2008; Kirilovsky 2015). The isolated samples, PBS and PSI, were diluted using the 0.75 M phosphate buffer and buffer A, respectively. OD at 577 nm for the isolated PBS sample was less than 0.5 and OD at *Q*_y_ maxima for isolated PSI sample was less than 1. A power study was made for each experiment to confirm the absence of annihilation.

Fluorescence was recorded from 590 to 860 nm and 0 to 400 ps (time range 2: TR2) and 0 to 1600 ps (time range 4: TR4). Each measurement consists of a sequence of 300 images, each of which results from a scan of 10 s. Image sequences were averaged and corrected for background and shading in HPD-TA software (Hamamatsu). These corrected datasets were sliced up into traces of 2 nm width, using Glotaran 1.5.1 (Snellenburg et al. 2012). The full width at half maximum (FWHM) of the instrument response function (IRF) was ≈ 7 ps with TR2 and ≈ 18 ps with TR4.

### Data analysis

The global and target analysis of time-resolved spectroscopy measurements aims to solve an inverse problem of decomposing time-resolved spectra into the superposition of contributions from different components

1 $$\Psi \left( {t,\lambda } \right) = \mathop \sum \limits_{i = 1}^{n} C_{i} \left( {t,\theta } \right)E_{i} \left( \lambda \right)$$

where $n$ is the number of components in the system, ${C}_{i}\left(\mathrm{t},\uptheta \right)$ is the *i*-th time-dependent concentration profile described by non-linear parameters $\theta$, and ${E}_{i}(\lambda )$ is the *i*-th spectrum. This problem is solved by estimation of the unknown parameters $\theta$ and $E$ with the Variable Projection algorithm (van Stokkum et al. 2004; Mullen and van Stokkum 2009).

#### Global analysis

In global analysis, the concentration profile ${C}_{i}$ is modelled as the convolution of an exponential decay and $\mathrm{I}\mathrm{R}\mathrm{F}(t)$. The associated spectra are called Decay Associated Spectra (DAS) in this case, then the formula (1) is given by

$$\Psi \left( {t,\lambda } \right) = \sum\limits_{i = 1}^{n} {e^{{ - k_{i} t}} *{\text{IRF}}\left( t \right) \cdot {\text{DAS}}_{i} \left( \lambda \right)}$$

where * indicates the convolution. ${k}_{i}$ are the decay rates, which are the inverse of the fluorescence lifetime (${\tau }_{i}$). Negative and positive amplitudes of the DAS correspond to rise and decay of emission, respectively, and indicate energy transfer from the donor to the acceptor (Holzwarth 1996).

#### Target analysis

In the case of target analysis, the concentration profiles ${\varvec{C}}\left( t \right) = \left[ {C_{1} \left( t \right),C_{2} \left( t \right), \ldots ,C_{n} \left( t \right)} \right]^{T}$ are the solutions of parametric first-order ordinary differential equations

$$\frac{{\text{d}}}{{{\text{d}}t}}{\varvec{C}}\left( t \right) = K{\varvec{C}}\left( t \right) + {\text{IRF}}\left( t \right){\varvec{j}}$$

with the initial condition

$${\varvec{C}}\left( {t = - \infty } \right) = 0$$

The non-zero off-diagonal element ${k}_{pq}$ in the coefficient matrix $K$, is the energy transfer rate from component *q* to component *p*, and diagonal element ${k}_{pp}$ represents the decay of component *p* caused by fluorescence or trapping. The initial condition ${\varvec{j}} = \left[ {j_{1} , j_{2} , \ldots ,j_{n} } \right]^{T}$ represents the initial excitation of each component.

In target analysis, the spectra ${E}_{i}$ are called the Species Associated Spectra (SAS), which reflect the real spectrum from each compartment (pigment pool). In streak measurements, the SAS represent emission, hence, non-negativity constraints are imposed in the optimization process. When a sequential irreversible kinetic scheme with increasing lifetimes is used, the SAS are called Evolution Associated Spectra (EAS).

#### Residual analysis, the singular vector decomposition (SVD)

For residual analysis, a singular vector decomposition (SVD) is applied to the matrix of residuals. The residual matrix ($res$) can be written as

$${\text{res}}\left( {t,\lambda } \right) = \mathop \sum \limits_{i = 1}^{m} {\text{lsv}}_{i} \left( t \right) \cdot s_{i} \cdot {\text{rsv}}_{i} \left( \lambda \right)$$

All time-dependent information is contained in the left singular vectors (${\text{lsv}}$), while right singular vectors (${\text{rsv}}$) contain information corresponding to wavelength $\lambda$. The ${s}_{i}$ are the singular values which reflect the relative importance of this term in the matrix. The ${\text{lsv}}$ and ${\text{rsv}}$ associated to the largest singular value show the main trends of the residual matrix in the time or wavelength dimension.

#### Simultaneous target analysis

To combine different experiments, a simultaneous target analysis was performed for selected datasets in each type of experiment. The simultaneous target model works as

$$\left[ {\begin{array}{*{20}c} {\Psi_{1} \left( {t,\lambda } \right)} \\ {\Psi_{2} \left( {t,\lambda } \right)} \\ \vdots \\ {\Psi_{m} \left( {t,\lambda } \right)} \\ \end{array} } \right] = \left[ {\begin{array}{*{20}c} {C_{1} \left( t \right)} \\ {\alpha_{2} C_{2} \left( t \right)} \\ \vdots \\ {\alpha_{m} C_{m} \left( t \right)} \\ \end{array} } \right] \cdot {\text{SAS}}\left( \lambda \right)$$

where $m$ is the number of datasets, ${C}_{i}$ is the concentration matrix of experiment *i* as explained in the previous section. The $\mathrm{S}\mathrm{A}\mathrm{S}$ matrix is shared by all the datasets. For each extra dataset, a scaling number $\alpha$ is necessary to describe the relative size in different measurements.

## Results and discussion

### Isolated systems

#### PSI-enriched fraction

**Fig. 2:**
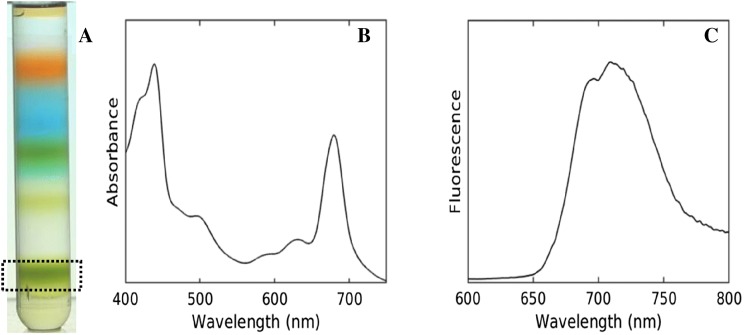
Characterization of isolated PSI-enriched fraction. **a** Sucrose density gradient centrifugation showing fractionation profile of n-dodecyl-β-d-maltoside(DM)-solubilized thylakoids. The sucrose density gradient contained 0.05% of DM (Watanabe 2014). The dotted boxed layer was used as the PSI-enriched fraction for further experimentation. Room temperature absorbance spectrum (**b**) and emission spectrum with 400 nm excitation (**c**)

The room temperature steady-state absorption and fluorescence spectra of the isolated PSI-enriched fraction from sucrose density gradient isolation are shown in Fig. 2a. The absorption spectrum (Fig. 2b) reveals the Chl *aQ*_y_ absorption maximum at 679 nm, which is typical for PSI (Andrizhiyevskaya et al. 2002). The fluorescence emission (Fig. 2c) at 400 nm excitation, shows a maximum at 710 nm with a shoulder at 693 nm, suggesting the predominant presence of PSI complexes in the isolated sample. To further characterize the isolated sample and to investigate the energy transfer and trapping processes, time-resolved fluorescence measurements were performed with a streak-camera system. Figure S1(A) shows the two-dimensional streak image obtained upon 400 nm excitation of the PSI-enriched isolated fraction.

**Fig. 3:**
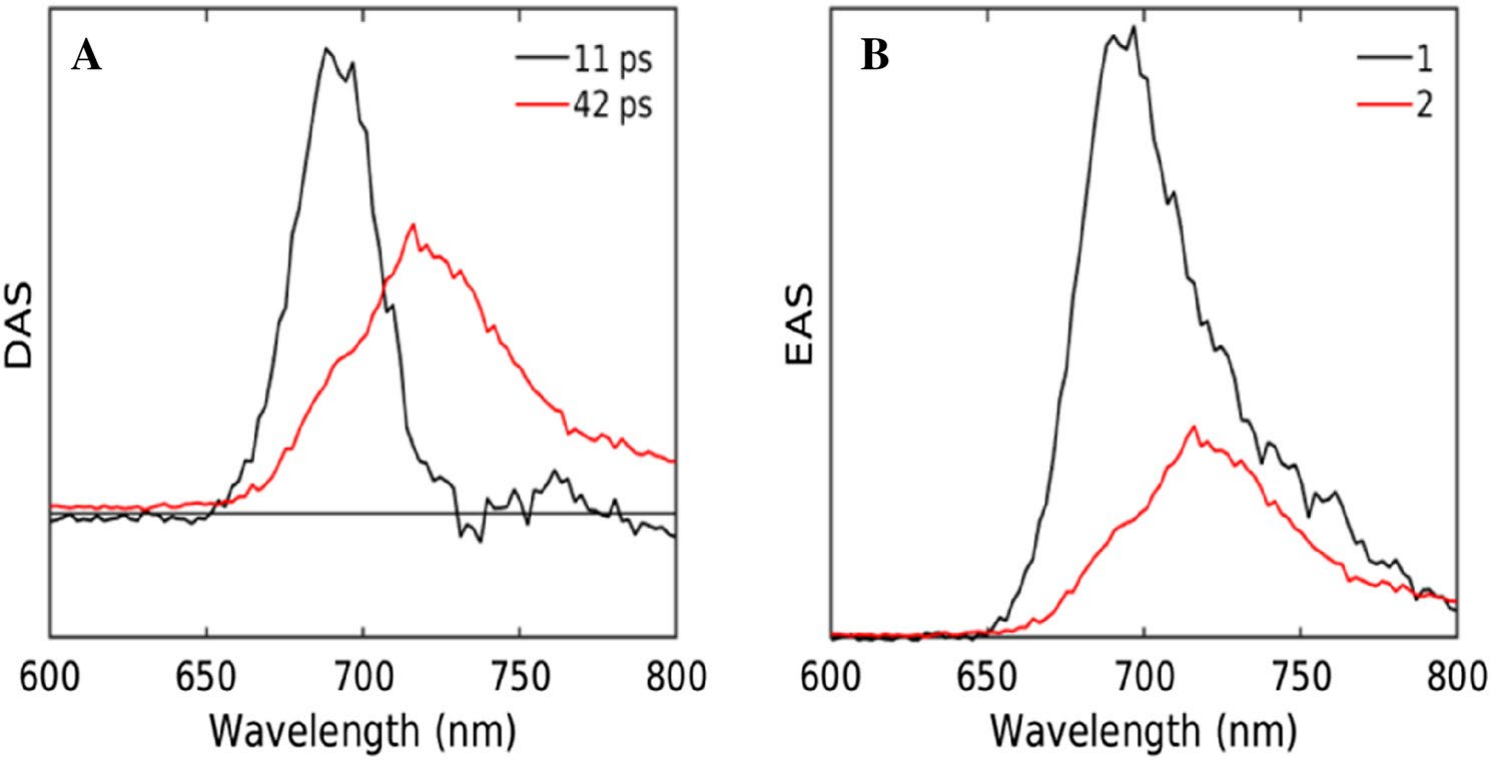
Estimated DAS (**a**) and EAS (**b**) upon 400 nm excitation of the PSI-enriched fraction

To describe the streak image quantitatively, global analysis was employed, which resulted in the DAS and EAS as shown in Fig. 3a, b. Two components were needed to adequately fit the data (Fig. S 1B, C). The 11 ps DAS has a positive peak at 690 nm and negative amplitudes around 720 nm. These reflect the EET between two pools of bulk and red Chl of PSI, although it also contains some decay as indicated by the net loss of amplitude. The 42 ps DAS is a pure decay component and represents trapping of the equilibrated PSI. The black EAS (Fig. 3b) represents the emission spectrum immediately after the excitation, which evolves into the red EAS.

#### Isolated phycobilisomes

**Fig. 4:**
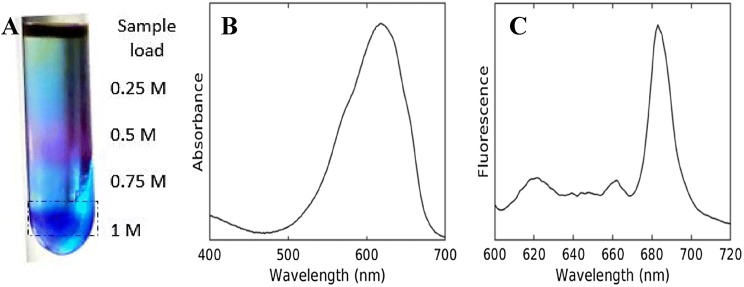
Isolation of PBS complexes and their spectral characterization. **a** The dark blue fraction resolved by the sucrose density gradient centrifugation, used as the sample for further characterization. **b** Absorption spectrum of the isolated PBS band at room temperature. **c** Steady-state fluorescence emission spectrum recorded at 77 K using excitation at 577 nm

Phycobilisomes isolated from the *Anabaena* filaments using sucrose density gradient centrifugation were characterized by steady-state absorption and fluorescence spectroscopy. The dark blue PBS band at around 1 M of the gradient (Fig. 4a, dotted box) shows an absorption maximum at 620 nm (Fig. 4b), which is typical for isolated PBS (Jallet et al. 2014). The low-temperature fluorescence emission spectrum (Fig. 4c) shows some emission in the PC region (≈ 630 nm) and a maximum at 683 nm, indicating efficient EET to the terminal emitter and also the functional intactness of the isolated PBS.

**Fig. 5:**
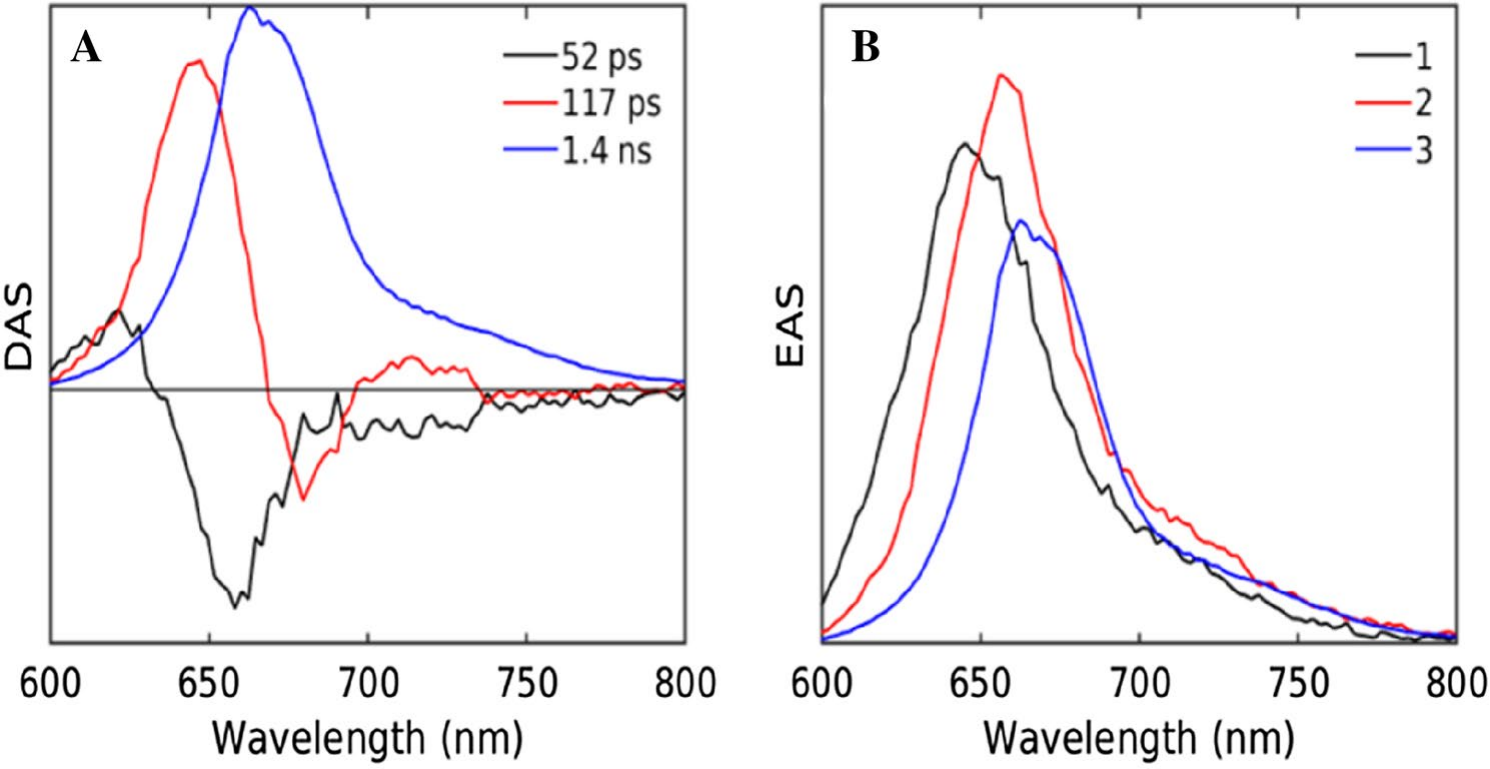
Estimated DAS (**a**) and EAS (**b**) upon 400 nm excitation of the isolated PBS fraction

Figure 5 shows the DAS and EAS estimated from the global analysis of the streak data of isolated PBS. It must be noted that the information obtained from the 400 nm excitation of this particular dataset is limited due to the non-selective excitation. Three components were needed to adequately fit the data (cf. Fig. S 2B, C). The 52 ps DAS shows a decay at 635 nm and a rise at 660 nm, reflecting EET from the PC to APC660. The 117 ps DAS with a rise near 680 nm suggests EET within the core. The fully equilibrated PBS decays with a lifetime of 1.4 ns.

#### Intact filaments

The fluorescence decay of the intact filaments was measured with 400 and 577 nm excitation, exciting preferentially (90%) the Chls of the PSs and the PBS, respectively. The streak images are shown in Fig. S 3A,B.

**Fig. 6:**
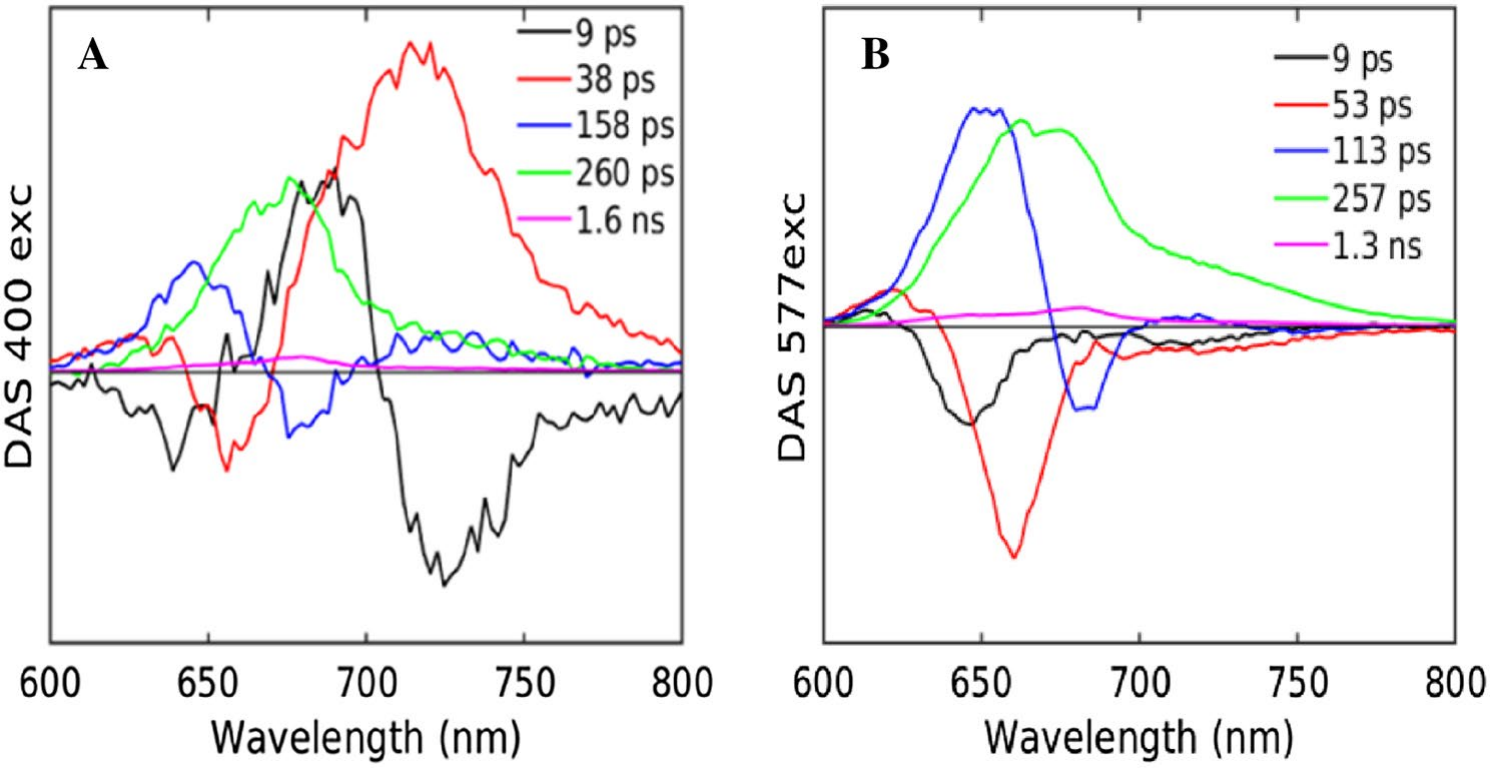
Estimated DAS upon 400 (**a**) or 577 (**b**) nm excitation of intact filaments at RT. Estimated lifetimes are collated in Table 1

The DAS and EAS estimated from a simultaneous global analysis of the two time-ranges of fluorescence data with 577 or 400 nm excitation are shown in Figs. 6 and S 4. The simultaneous global analysis of 577 nm excitation required five lifetimes (Fig. 6b). The 9 ps DAS has a positive peak at 620 nm and negative peak at around 650 nm, thus indicating the EET within the rods. The 53 ps DAS shows a positive peak at around 630 nm and a negative peak at 660 nm, reflecting the EET from rod to core. The 113 ps DAS suggests EET within the core and with Chls of the PS. Finally, the last two components are entirely positive in nature, with lifetimes of 257 ps (PSII trapping) and 1.3 ns (non-transferring PBS). The 400 nm excitation data also were best fitted with 5 lifetimes (Fig. 6a). The 9 ps DAS shows equilibration within the rods along with EET in PSI from bulk to red Chls. The 38 ps DAS shows primarily the charge separation in PSI and also reflects the EET from rod to core. The third component of 158 ps reflects EET within the core. The two final lifetimes of 260 ps and 1.6 ns are similar to those with 577 nm excitation and can be interpreted as trapping by PSII and non-transferring PBS, respectively. These complicated DAS can only be interpreted using a kinetic model. The fit result for the whole filament global analysis is shown in Figure S 5.

The estimated lifetimes of all experiments were consistent (Table 1). Thus, in order to resolve each of the chromophore groups present within the intact filaments, and to gain further insight into the EET dynamics, a functional compartmental model must be developed.

**Table 1:** Comparison of lifetimes (in ps) estimated from global analysis of PSI, PBS and intact filaments

Sample\lifetime	*τ*1	*τ*2	*τ*3	*τ*4	*τ*5
PSI	11	42			
PBS		52	117		1400
Filaments, 400 nm exc	9	38	158	260	1600
Filaments, 577 nm exc	9	53	113	257	1300

#### Target analysis

The target model was designed based on the results obtained from the global analysis of the isolated systems and the intact filaments, and was inspired by the literature reviewed in the introduction. Both intact and isolated systems can be described by a superposition of the dynamics of the chromophore groups (compartments). It was taken into consideration that the varying excitations among the intact cells would emphasize a particular aspect of the system. For instance, with 400 nm excitation of the intact cell, the fast kinetics within the PSI will be probed primarily, and further the PSI-enriched isolate will provide robustness in the precise estimation of the fast PSI kinetics and also to the spectral shapes. On the other hand, the 577 nm excitation dominantly excites the PC of the PBS and therefore these data allow for a reliable estimation of EET within the PBS. Therefore a step-wise approach of systems modelling was adapted here, where a minimal reasonable model was tested in each isolated system and the information acquired from these systems was used as a priori information for the whole cell modelling. Additionally, reasonable spectral assumptions had to be used to resolve the congested SAS.

**Fig. 7:**
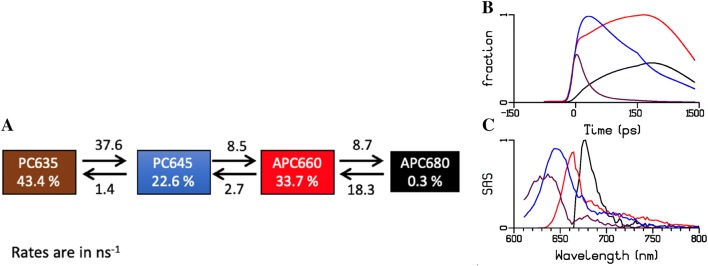
Target analysis of the time-resolved fluorescence data of isolated PBS with 400 nm excitation. **a** Kinetic scheme for the target analysis showing each compartment (coloured boxes) representing a pool of pigments The energy transfer rates (ns^−1^) between these compartments and their concentration profiles (**b**) and SAS (**c**) were estimated from the fitting. Key: PC635 (maroon), PC645 (blue), APC660 (red), APC680 (black). The time axis in B is linear until 150 ps and logarithmic thereafter. The relative percentage of excitation of each pigment pool is also shown within the boxes

The PBS system can be described by a kinetic scheme with four compartments (Tian et al. 2011, 2012), namely: PC1, PC2, APC1, APC2. The estimated SAS show maxima at approximately 635, 645, 660 and 678 nm (Fig. 7). The maroon and blue SAS correspond to the blue and red-shifted phycocyanin pigments. The red and the black SAS represent APC bulk and the APC terminal emitter. Henceforth, we will designate the PBS compartments as PC635, PC645, APC660, and (in keeping with the literature) APC680.

The quality of the fit is shown in Fig. S 6. The concentration profiles (Fig. 7c) show the evolution of the populations of the compartments. Note that the rod to core equilibration is relatively slow. The intracore equilibration between APC660 and APC680 is highly simplified, since many time scales are present with intradisc, interdisc and intercylinder equilibration, and the estimated rates represent an average. Table S 2 shows the amplitude matrix for the proposed target analysis of isolated PBS.

**Fig. 8:**
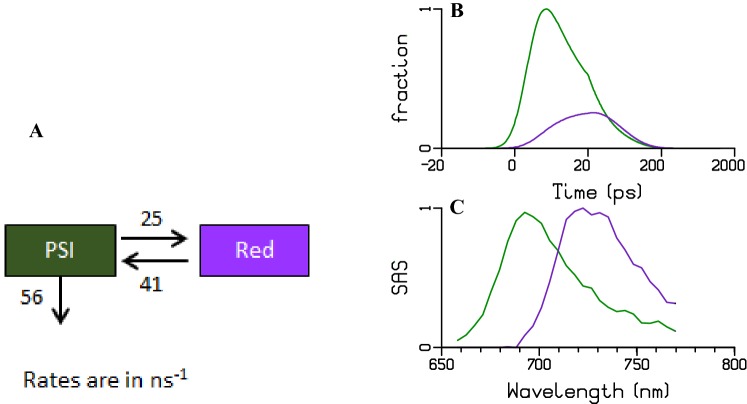
Target analysis of the time-resolved fluorescence data of PSI with 400 nm excitation, rates in ns^−1^. **a** Kinetic scheme **b** Total concentration and **c** estimated SAS. Key: bulk Chl of PSI (dark green), Red Chl of PSI (purple). The time axis in B is linear until 20 ps and logarithmic thereafter

PSI can be adequately described with two compartments, a bulk compartment (where the charge separation takes place) and a red-emitting Chl compartment (which is in equilibrium with the bulk Chl and emits at lower energy). Using the kinetic scheme from Fig. 8a, we obtained the SAS and concentration profiles shown in Fig. 8. The quality of the fit is good, as can be seen in Fig. S 7. The dark green and purple SAS represent the bulk Chls and red Chls of PSI with emission maxima at 695 nm and 718 nm, respectively. Table S 1 shows the relative excitation of each PSI pigment pool. The DAS of PSI can be interpreted with the help of the amplitude matrix, elaborating the population and depopulation of each compartment at each lifetime, cf. Table S 1.

**Fig. 9:**
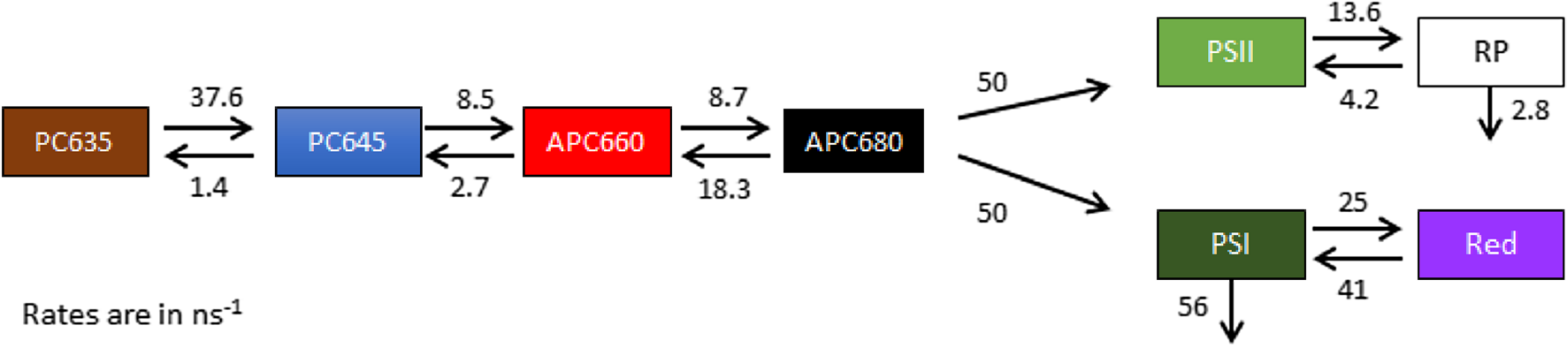
Estimated kinetic model of the Megacomplex 1 consisting of PBS-PSII-PSI from simultaneous target analysis of all in vitro and in vivo datasets, rates in ns^−1^. Fluorescence decay rates (0.71 ns^−1^) have been omitted for clarity. Key: PC635 (maroon), PC645 (blue), APC660 (red), APC680 (black), PSII Chl a (green), PSI Chl a (dark green) and PSI “Red Chl” (purple)

The overall kinetic scheme for the cell consists of three different “megacomplexes”, with each megacomplex consisting of several compartments. Megacomplex 1 involves PBS-PSII-PSI as PBS is well known to feed energy to both PSs, i.e. PBS coupled to both PSI and PSII (Liu et al. 2013; Acuna et al. 2018a; Tian et al. 2011). PSII is known to be best described with a core which is in equilibrium with a non-radiative radical pair (RP) (Tian et al. 2013). Megacomplex 2 involves non-transferring PBS. And lastly, Megacomplex 3 involves non-transferring rods (PC635, PC645). Despite using fresh samples for each measurements and cautious sample handling, a relatively small amount of free PBS or free rods (5–12%) was observed [reported before in (Acuna et al. 2018b)], which was also taken into consideration for our kinetic modelling, as Megacomplex 2 and 3. A simultaneous target analysis with eight best datasets (four datasets from the cell measurement, and two from each of the isolated systems measurements) were performed using the kinetic model shown in Fig. 9. The overall target model contains 15 rate parameters, 8 parameters for the ratio of detached megacomplexes, ($8\times 2= 16$) parameters for excitations, 7 dataset scaling parameters, 52 IRF parameters, plus the SAS ($94\times 8=$ 752 conditionally linear parameters). Thus, with the target analysis, we aim to simultaneously fit the cyanobacterial datasets with a minimal kinetic model, and estimate the SAS. The non-radiative RP has a zero SAS and the PSII SAS were constrained to be zero below 655 nm. Essential to the target analysis are the spectral shapes and area of the estimated SAS (van Stokkum et al. 2004; Mullen and van Stokkum 2009). The initial input for the excitation vector for each compartment is based on the absorption spectrum and on a priori knowledge. In addition, the fit quality is judged based on the analysis of the residual matrix (Fig. S 8). Singular value decomposition of the residual matrix helped us to locate where misfit happens and thus improvement was needed in the time or wavelength domain.

**Fig. 10:**
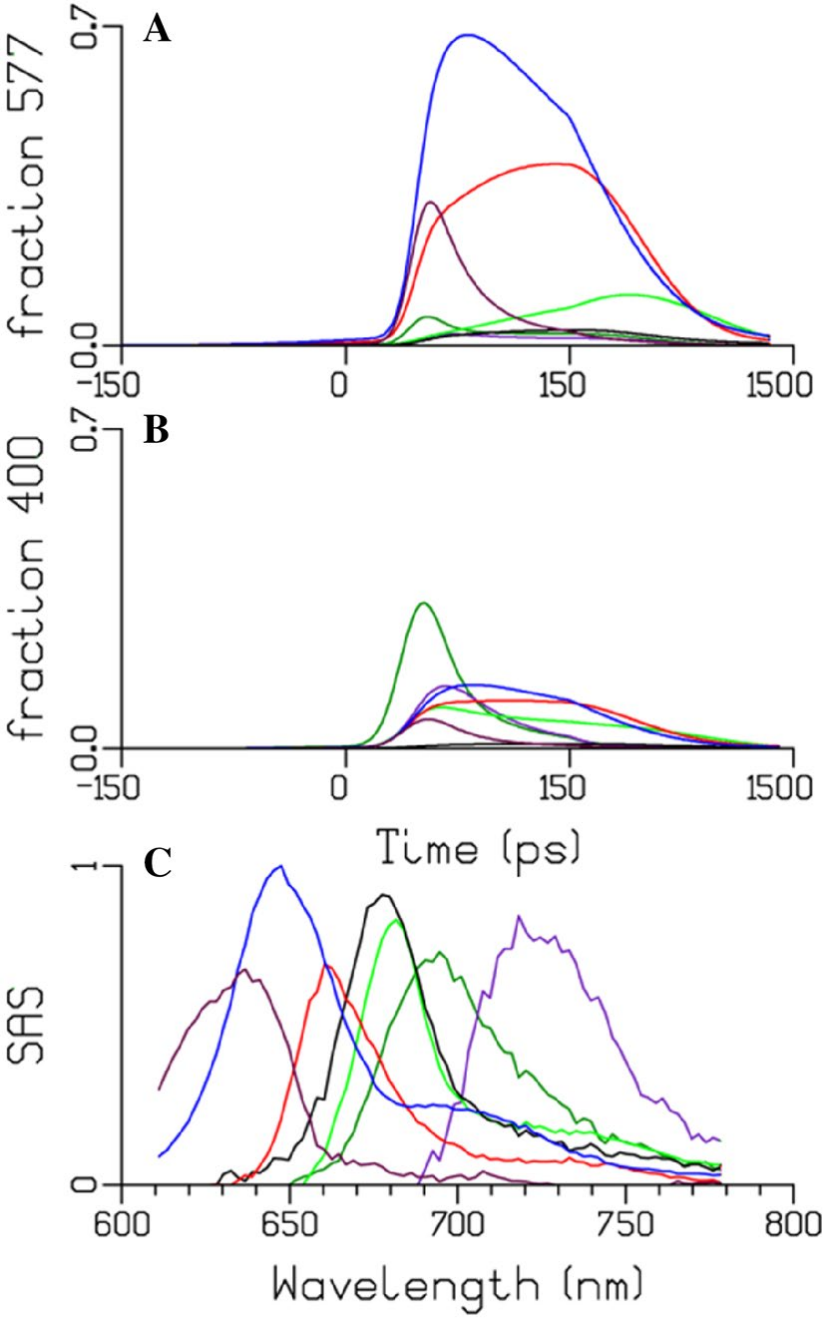
Estimated total concentrations after 577 (**a**) or 400 (**b**) nm excitation and SAS (**c**). Key: PC635 (maroon), PC645 (blue), APC660 (red), APC680 (black), PSII Chl a (light green), PSI Chl a (dark green) and PSI “Red Chl” (purple). The time axis in **a**, **b** is linear until 150 ps and logarithmic thereafter

The simultaneous target analysis using the kinetic model (Fig. 9) resolved seven well-interpretable SAS (Fig. 10) and the relative precision of most of the microscopic rate constants was 20% or better. They agree with the SAS estimated from the isolated systems (Figs. 7, 8). The maximum of the PSII Chl a (green) emission was at 681 nm. The resolved PSI and PSII SAS show a nice long vibrational Chl tail. The PSI SAS emission spectrum is clearly red shifted with respect to that of PSII. The fit quality of the target analysis is good, cf. Fig. S 8. The estimated fractions of each megacomplex are collated in Table 2.

**Table 2:** Estimated fractions of the three megacomplexes present in intact filaments upon simultaneous target analysis

Megacomplex	400 exc TR4 (%)	400 exc TR2 (%)	577 exc TR4 (%)	577 exc TR2 (%)
PBS-PSII-PSI	91	88	93	95
Non-transferring PBS	8	10	3	1
Non-transferring PC	1	2	4	4

The overall trapping of the excitation energy in PSI core occurs typically in 12–60 ps in cyanobacterial organisms and higher plants depending on the antenna size and the amount of red Chls (Croce et al. 2000; Wientjes et al. 2011; van Stokkum et al. 2013; Akhtar et al. 2018; Gobets et al. 2001). The estimated PSI trapping observed in our model is  ≈18 ps which is comparable with the previously observed lifetimes of  ≈16 and  ≈ 2 ps in *Synechocystis* and *Synechococcus* (Tian et al. 2011; Acuna et al. 2018a). The 18 ps PSI trapping seems to be a plausible average decay time considering the small amount of red Chl in a pool of  ≈100 bulk Chls (cf. Table S 5. Free energy calculations.). The equilibrium of the PSII bulk and radical pair compartments with a forward rate of 13.6/ns and a backward rate of 4.2/ns agree with previously estimated rates involving PSII dynamics with open RCs in *Synechococcus* and *Synechocystis* (Acuna et al. 2018a, b; Tian et al. 2011, 2013). The estimated rates of EET from the terminal emitter of PBS to PSs i.e. APC680 to PSII and PSI are 50/ns and 50/ns, respectively. Since the equilibration between the PBS rod and core is much slower, it is extremely difficult to estimate these faster rates. Therefore, we fixed the rate from APC680 to PSII to 50/ns based upon (Acuna et al. 2018a). The rate of APC680 to PSI was very similar, and ultimately we chose it to be 50/ns as well. Changing these rates to values between 35 and 120/ns did hardly affect the quality of the fit. However, when the rate from APC680 to PSI becomes smaller, e.g. APC680 to PSII and PSI 50/ns and 25/ns, respectively, the fit quality begins to deteriorate, cf. Fig. S 9. Megacomplexes from Synechocystis have been measured at 77 K (Liu et al. 2013) where it was suggested that the EET rate of APC680 to PSI was slower than that to PSII based upon a delayed rise of the emission observed at 720 nm. After 577 nm excitation (green in Fig. S 10), we also observe a delayed rise of the emission above 650 nm which can be attributed to the PBS energy funnel. With these congested SAS (Fig. 10c), EET rates cannot be inferred from the observed emission traces, but can only be estimated with the help of a target analysis. A likely consequence of the equal EET from PBS to PSI and PSII might be that PSI receives more excitations than PSII under natural light conditions. This is, on the one hand, because PSI is more abundant than PSII (Manodori and Melis 1986; Fujita 1997) and because of the larger intrinsic antenna of PSI, on the other. It can be expected, at least under state 1 conditions, that a fraction of PSI not connected to any PBS are still directly excited. Cyanobacteria possess a number of mechanisms to maintain excitation/redox balance and regulate electron transport, for example, via cyclic electron transport routes involving PGR5 or NDH1 (Mullineaux 2014). Thus, irrespective of the differences in the PBS structure, the EET is optimized and the energy funnelling distribution to both the PSs remains almost equal in both cyanobacterial organisms, *Anabaena* and *Synechocystis*. The importance of the APC680 to PSI EET is consistent with Dong and Zhao (2008) who established that state transitions are present in *Anabaena*. As a mechanism for the state 2 to state 1 transition (Acuña et al. 2018) proposed a deceleration of the APC680 to PSI EET rate.

Despite the spectral overlap between the SAS of the APC680 and PSII compartments, both could be well resolved, because of the presence of free PBS which provided information on APC680 (Figs. 7b, 10c). The rod to core equilibration is estimated to be 8.5/ns and 2.7/ns, indicating that this is the rate-limiting step in the overall transfer process within the PBS as discussed before in (Zhang et al. 1997; van Thor et al. 1998). Perhaps, this rate might also be influenced partly by the slow kinetics within the APC (Choubeh et al. 2018). The rates nearly agree with the results from van Stokkum et al. (2018). The thermodynamic calculations between the rod to core and within the core are shown in Table S 5. The equilibration within the rods of 38/ns is estimated to be the fastest in our kinetic model of PBS. The PC635 with a broad spectral emission can be considered as a contribution from two groups of chromophores (PC and PEC) commonly present in the distal rods of the PBS structure of *Anabaena* (Ducret et al. 1998). These two compartments could not be separated with our model. It must also be noted that the SAS of the fastest decaying species is sensitive to the relative absorption parameters. The amplitude matrices of the target analysis display the rise and decay times and decay associated amplitudes of the concentrations (Fig. 10a, b), and are shown and discussed near Table S 3 and Table S 4*.*

## Conclusion

We have arrived at a target model for EET and trapping in *Anabaena* cells (Fig. 9) that incorporates the target models for PBS (Fig. 7) and PSI (Fig. 8). The estimated kinetic parameters and SAS are realistic. The kinetic rates describing the EET from the PBS terminal emitter to the PSs still had to be fixed. In the future we aim to elucidate this EET further.

## Electronic supplementary material

Below is the link to the electronic supplementary material.

Supplementary file1 (DOCX 4157 kb)
