# Supplementary material for: Modelling excitation energy transfer and trapping in the filamentous cyanobacterium Anabaena variabilis PCC 7120
Source: Photosynth Res. 2020 Feb 19;144(2):261–72. doi: 10.1007/s11120-020-00723-0 (PMC7203589; doi:10.1007/s11120-020-00723-0)
Supplement: Supplementary file 1 — Supplementary file1 (DOCX 4157 kb) [file 11120_2020_723_MOESM1_ESM.docx]

### Modelling Excitation Energy Transfer and Trapping in the Filamentous Cyanobacterium *Anabaena variabilis* PCC 7120

Avratanu Biswas, Xinpeng Huang, Petar H. Lambrev, Ivo H.M. van Stokkum

### Supplementary material

**A**

**B**


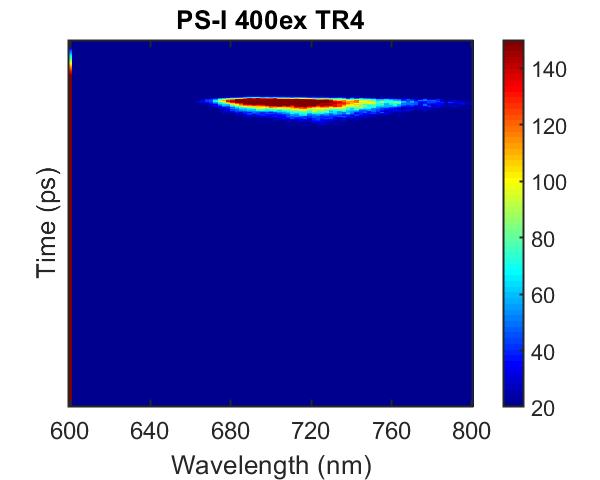

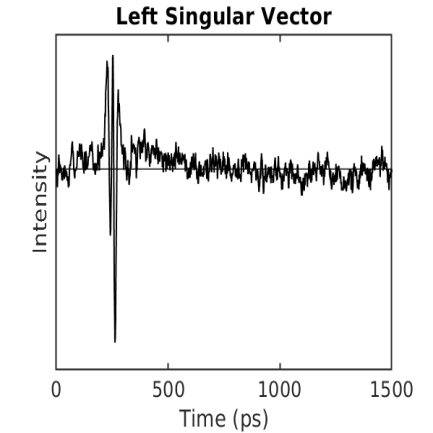

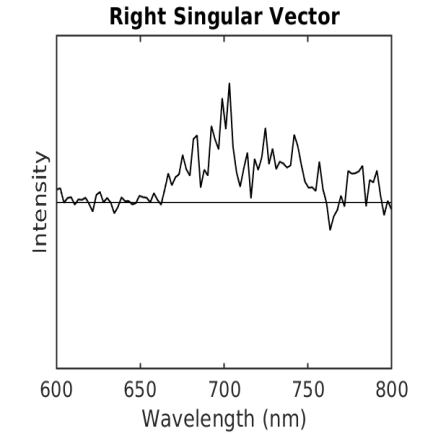


**C**

**C**

Figure S 1. (A) 2-D streak image of the PSI-enriched sample upon 400 nm excitation at time range 4 (0 to 1600 ps). SVD of the residual matrix after global analysis of the PSI-enriched sample (B) first LSV (C) first RSV.

**B**


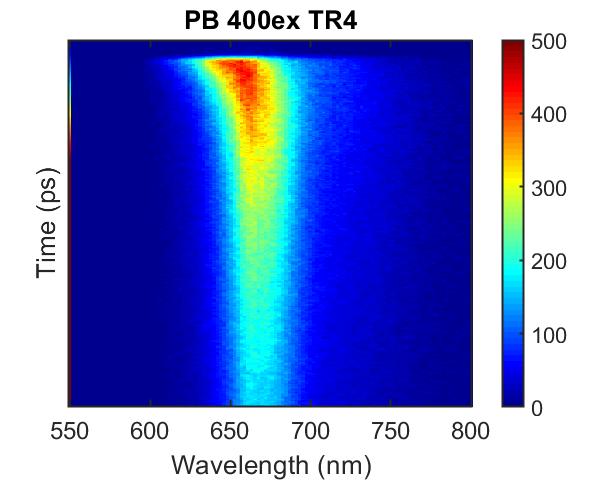

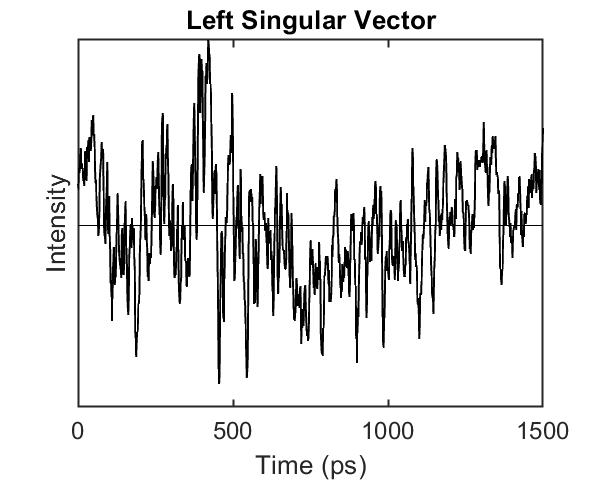

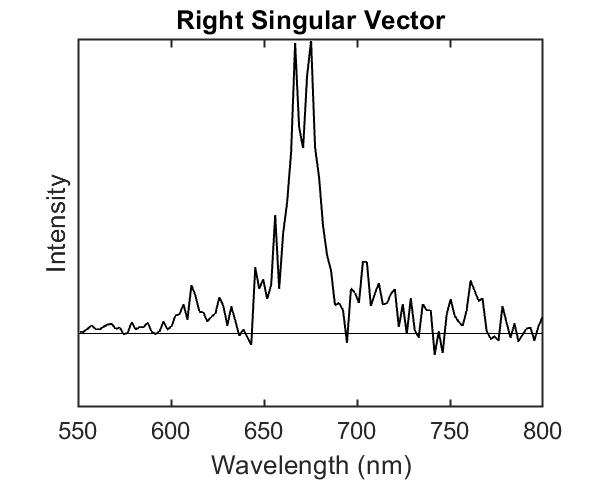


**C**

**A**

Figure S 2. (A) 2-D streak image of the isolated PBS sample upon 400 nm excitation at time range 4 (0 to 1600 ps). SVD of the residual matrix after global analysis of the isolated PBS sample (B) first LSV (C)first RSV


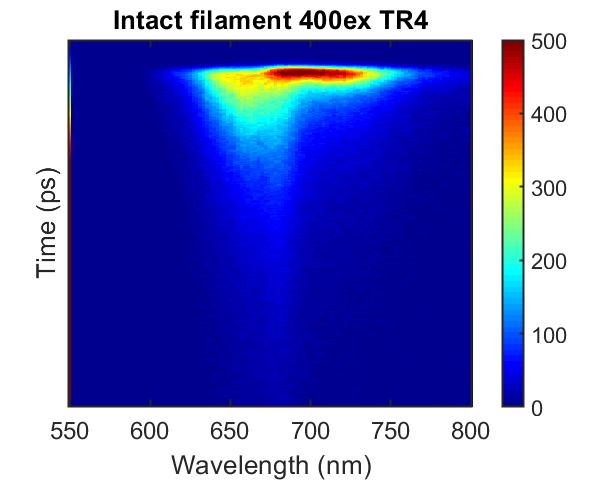

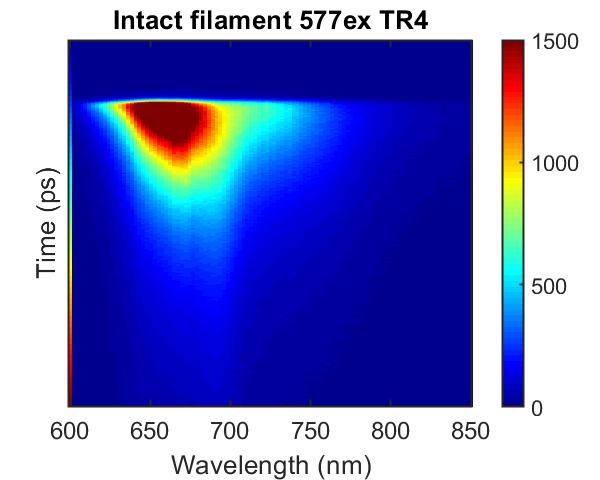


**A**

**B**

Figure S 3. (A) 2-D streak image image of the intact filaments upon (A) 400 nm and (B) 577 nm excitation at time range 4 (0 to 1600 ps).


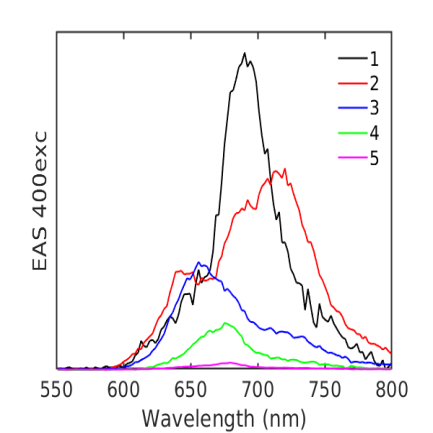

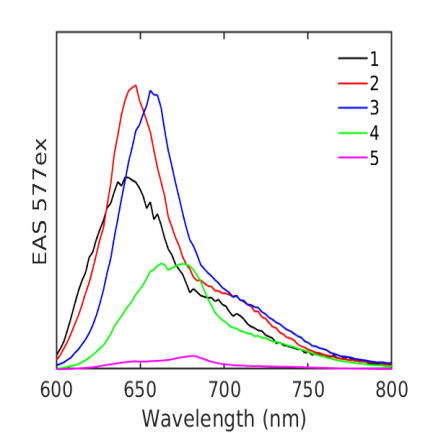


**B**

**A**

Figure S 4. Estimated EAS upon 400 (A) or 577 (B) nm excitation of intact filaments at RT. Estimated lifetimes are collated in Table 1.


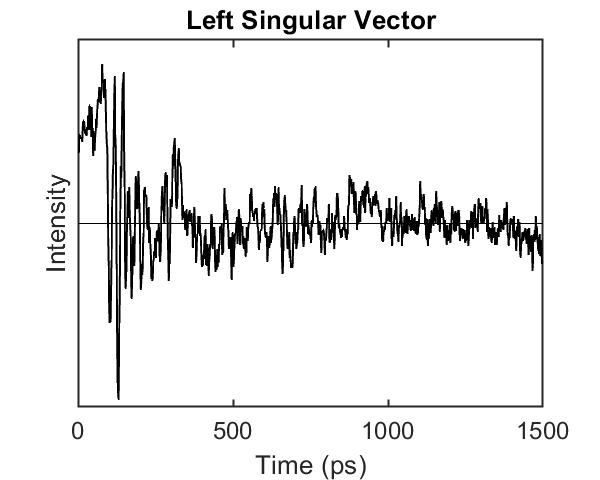

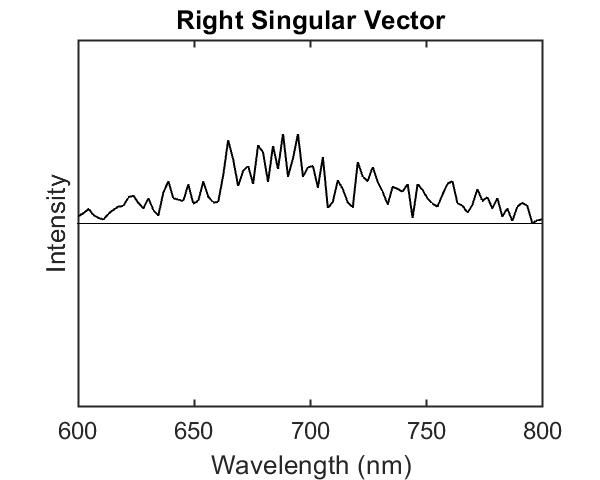


**A**

**B**


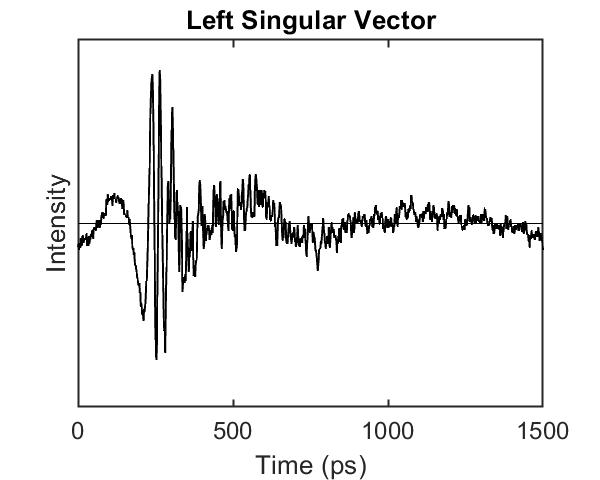

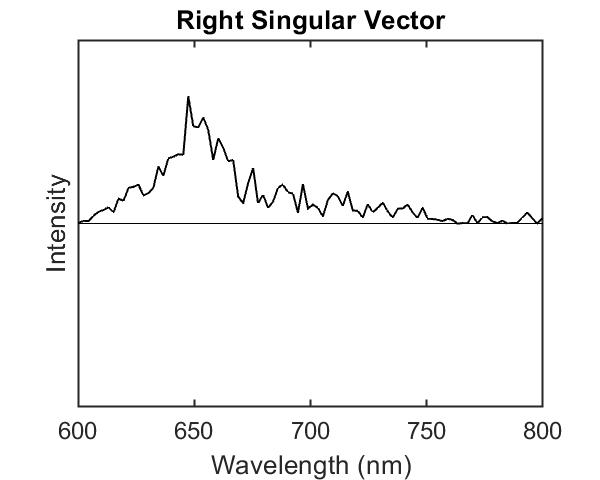


**C**

**D**

Figure S 5. Singular value decomposition of the residual matrix after applying global analysis to intact filaments showing first left and right singular vectors upon 400 nm and 577 nm excitation.


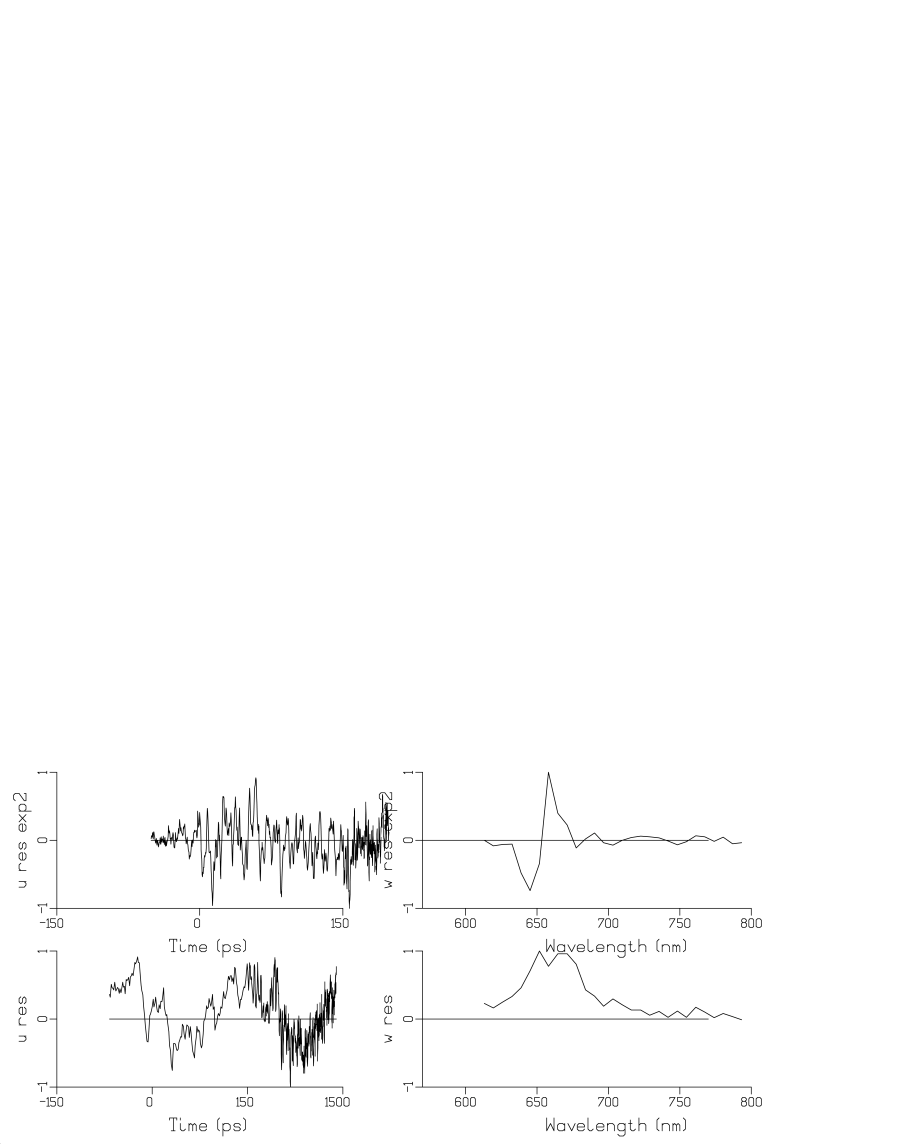


**A**

**C**

**B**

**D**


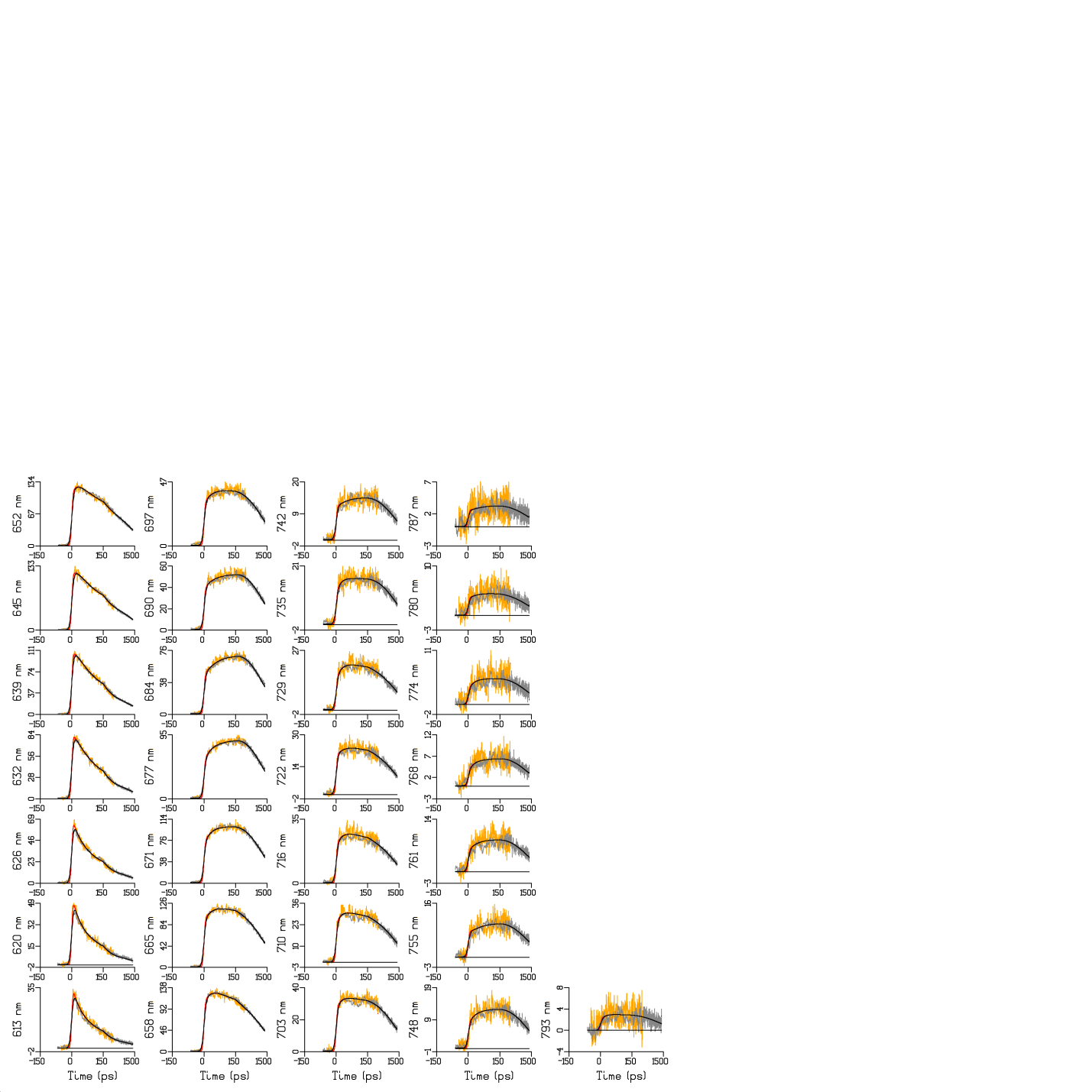


**E**

Figure S 6. Fit quality for the target model of PBS. First left and right singular vectors of the residual of TR2 data (A,B) and TR4 data (C,D). (E) Emission at 29 wavelengths (indicated in the ordinate label) after 400 nm excitation of PBS at RT. Key: TR4 (grey), TR2 (orange). Black and red lines indicate the simultaneous target analysis fit. The time axis is linear until 150 ps and logarithmic thereafter.


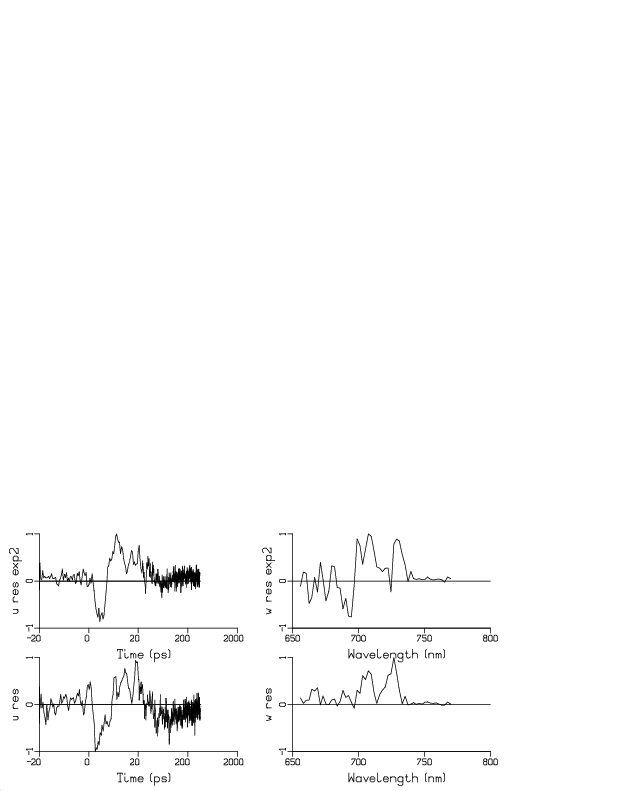


**C**

**A**

**B**

**D**


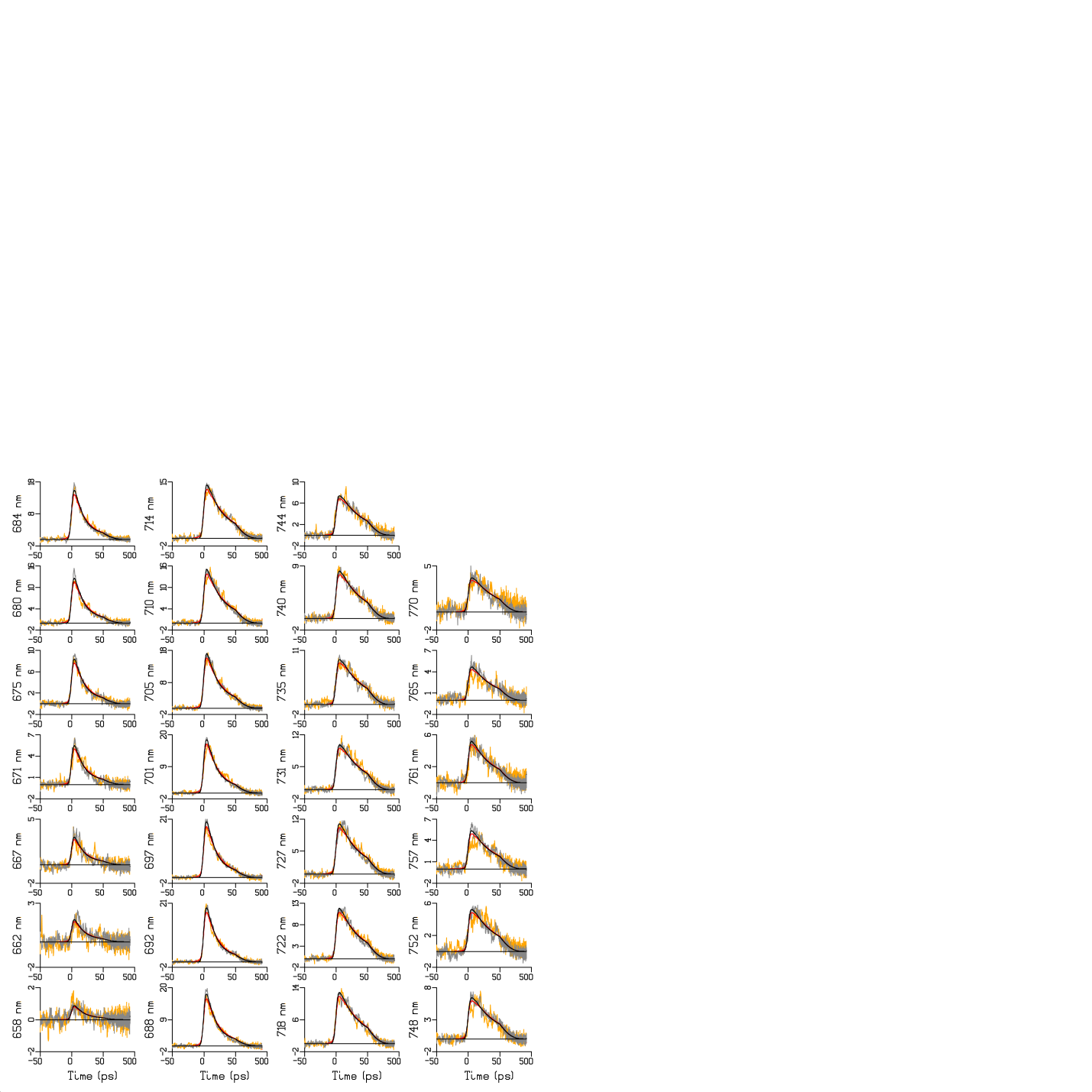


**E**

Figure S 7. Fit quality for the target model of PSI. First left and right singular vectors of the residual matrix of the TR2 data1 (A,B) and data2 (C,D). (E) Emission at 27 wavelengths (indicated in the ordinate label) after 400 nm excitation of PSI at RT. Key: TR2 data1 (grey), TR2 data2 (orange). Black and red lines indicate the simultaneous target analysis fit. The time axis is linear until 50 ps and logarithmic thereafter.

**E**

Figure S 8. Fit quality for the target model of the intact cell. First left and right singular vectors of residual matrix of the 400 (A,B) and 577 (C,D) nm excitation data. Key: TR2, red and TR4, black. (E) Selected traces of cell emission at 42 wavelengths (indicated in the ordinate label) at RT. Key: intact cells after 400 nm excitation recorded at TR4 (grey), TR2 (orange); intact cells after 577 nm excitation recorded at TR4 (cyan), TR2 (green); Dark colors indicate the simultaneous target analysis fit. The time axis is linear until 150 ps and logarithmic thereafter. Rms error of the fit is 0.626.

Figure S 9. Fit quality with an alternative target model of the intact cell, where the EET rate from APC680 to PSI has been fixed at 25/ns instead of 50/ns. First left and right singular vectors of residual matrix of the 400 (A,B) and 577 (C,D) nm excitation data. Key: TR2, red and TR4, black. Rms error of the fit is 0.634, which is 1% worse than in Figure S 8. Comparing to Figure S 8, we note that the u res in panel C now shows a positive trend from ≈30-≈100 ps, in combination with a peak around 680 nm in w res in panel D.

Figure S 10. Comparison of selected TR2 traces of cell emission at twelve wavelengths (indicated in the ordinate label) at RT. Key: intact cells after 400 (orange) or 577 (green) nm excitation, PSI after 400 nm excitation (brown). Dark colors indicate the simultaneous target analysis fit. The time axis is linear. To improve the signal to noise ratio (cf. Figure S 8), four traces have been added.

## Amplitude matrices of the target analyses

Table S 1. Amplitude matrix of PSI at RT upon 400 nm excitation. Color code of the species corresponds to that in the kinetic scheme, Figure 8A.

From the Table S 1, the 10 ps lifetime shows a positive value 0.756 of PSI Bulk (corresponding to the depopulation of the compartment) and negative value -0.335 of PSI red (corresponding to the rise of the population of the compartment), reflecting the equilibration between the two pools of pigments in the PSI core. The sum of the positive and negative amplitude is 0.421, which indicates that part of the trapping already takes place on the 10 ps time scale. Most of the trapping occurs on the 42 ps time scale from the equilibrated bulk and red Chl compartments of PSI.

Table S 2. Amplitude matrix of the PBS at RT upon 400 nm excitation. Color code of the species corresponds to that in the kinetic scheme, Figure 7A. Highlighted amplitudes are discussed in the text.

When PBS is excited with 400 nm (Table S 2), the PC635 and PC645 compartments equilibrate with the fastest lifetime of 25 ps (with transfer of population from PC635 to PC645, yellow highlight). The rod to core equilibration occurs on a time scale of 98 ps (orange highlight), as evidenced by the decay of PC645 (amplitude 0.556) and the rises of APC660 and APC680 (negative amplitudes -0.294 and 0.290). Within the core, the APC660 and APC680 compartments equilibrate on the 35 ps time scale (green highlight). The equilibrated isolated PBS decays with 1.4 ns, which is the natural lifetime of the pigments.

Table S 3. Amplitude matrix of the PBS-PSI-PSII at RT upon 577 nm excitation. Color code of the species corresponds to that in the kinetic scheme, Figure 9A. Highlighted amplitudes are discussed in the text.

Analogous to the isolated PBS, the 25 ps time constant mainly involves the decay and rise in population (highlighted in blue) between the PC635 and PC645 compartments. Subsequently, the rod to core equilibration time constant is 69 ps (highlighted in orange). The 35 ps equilibration between APC660 and APC680 is not observed in a PBS-PSI-PSII megacomplex because of the fast trapping of APC680 excitations by the two PSs, cf. Figure 9. Finally, the equilibrated PBS decays with a time constant of 200 ps (highlighted in maroon). The PSI bulk Chl and red Chl equilibrate on a time scale of 10 ps (highlighted in yellow) and the decay of PSI takes place at 42 ps timescale (highlighted in purple). These lifetimes are well observable due to the direct excitation of the PSI which is approximately 11%. In PSII with open RC, several time constants contribute to the charge separation dynamics, the main time constants are 52, 69 and 200 ps (highlighted in light green), and finally the equilibrated PSII and RP compartments decay with 447 ps (highlighted in dark green). The amplitudes observed on the time scale of 8.3 ps are all very small. They mainly represent the EET from PBS to PSI and PSII, which is very difficult to observe directly.

Table S 4. Amplitude matrix of the PBS-PSI-PSII at RT upon 400 nm excitation. Color code of the species corresponds to that in the kinetic scheme, Figure 9A. Highlighted amplitudes are discussed in the text.

By definition, all observed lifetimes are independent of the wavelength of excitation, but the amplitudes differ, cf. Table S 3 and Table S 4. With 400 nm light, the direct excitation of PSI and PSII are 70.1% and 8.4%, respectively. The remaining direct excitation (21.5%) is distributed over the PBS compartments. Qualitatively, the same dominant amplitudes are present, indicated with the same highlight colors.

## Free energy calculations

From the estimated rate constants indicated in the kinetic scheme of Figure 9, the free energy difference between two compartments that are in equilibrium can be computed using the definition of Free energy change in Table S 5. The enthalpy change equation gives us the estimated energetic contribution from the difference between the SAS maxima of the two equilibrated compartments (645 nm, 660nm, 678nm, 695nm, 718nm for PC645, APC660, APC680, PSI bulk and PSI red respectively). With the Gibbs free energy equation, see the definition of estimated entropy change in Table S 5, the estimated entropy difference i.e. ratio of number of pigments, between the equilibrated compartments could be calculated. The computed entropy difference is considered based on its rough consistency with previous literature values. In the case of the chromophores present in the rods , it is always speculated that the size of the rod is dependent on the growth conditions of the growing organism (Singh et al. 2015), thus the chromophore number for the PC (≈153 PC645 chromophores) taken into account is the best possible approximation. In our proposed model, between PC645 and APC660, the estimated chromophore ratio is 1.7 (which is in close approximation to the previously cited literature (Ducret et al. 1996; Jallet et al. 2014)). The APC core of *Anabaena*  has two flanking cylinders, thus we expect an additional ≈24 APC660 (in total 90 APC660) chromophores in the core of *Anabaena* when compared to the 66 APC660 chromophores in *Synechocystis* (as reported in (van Stokkum et al. 2018)) contributing to the entropy difference between the two APC compartments*.* Thus, the APC660 to APC680 ratio equals $\frac{90}{6}=15$, which serves as the integral parameter to compute the equilibration rate for optimizing the proposed model. Finally, based on an approximation of 96 Chls in a PSI monomer, our estimated pigment ratio between PSI bulk and PSI red is 15, hence giving an estimate of ≈6 red chlorophylls in the PSI red compartment, which seems to be a plausible biophysical parameter (Jordan et al. 2001).

| **Quantity** | **Definition** | **PC645↔APC660** | **APC660↔APC680** | **PSI Bulk↔PSI Red** |
| --- | --- | --- | --- | --- |
| Free energy change | $\Delta G=k_{B}T\ln(\frac{k_{forward}}{k_{backward}})$ | 29.6 meV | -19.2 meV | -12.8 meV |
| Enthalpy change | $\Delta H=hv_{1}-hv_{2}$ | 43.7 meV | 49.9 meV | 57.1 meV |
| Estimated entropy change | $T\Delta S=k_{B}T\ln\left( \frac{N_{1}}{N_{2}} \right)=\Delta H-\Delta G$ | 13.9 meV | 69.1 meV | 69.9 meV |
| Estimated Pigment ratio | $\frac{N_{1}}{N_{2}}$ | 1.7 | 15 | 15 |

Table S 5. Free energy calculations.( using k_B_T ≈ 25.85 meV )

# References

Ducret A, Sidler W, Wehrli E, Frank G, Zuber H (1996) Isolation, characterization and electron microscopy analysis of a hemidiscoidal phycobilisome type from the cyanobacterium Anabaena sp. PCC 7120. Eur J Biochem 236 (3):1010-1024.

Jallet D, Thurotte A, Leverenz RL, Perreau F, Kerfeld CA, Kirilovsky D (2014) Specificity of the cyanobacterial orange carotenoid protein: influences of orange carotenoid protein and phycobilisome structures. Plant Physiol 164 (2):790-804.

Jordan P, Fromme P, Witt HT, Klukas O, Saenger W, Krauss N (2001) Three-dimensional structure of cyanobacterial photosystem I at 2.5 angstrom resolution. Nature 411 (6840):909-917.

van Stokkum IHM, Gwizdala M, Tian L, Snellenburg JJ, van Grondelle R, van Amerongen H, Berera R (2018) A functional compartmental model of the Synechocystis PCC 6803 phycobilisome. Photosynthesis Research 135 (1):87-102.

Singh NK, Sonani RR, Rastogi RP, Madamwar D (2015) The phycobilisomes: an early requisite for efficient photosynthesis in cyanobacteria. EXCLI J 14:268-289.
